# Conveniently dependent or naively overconfident? An experimental study on the reaction to external help

**DOI:** 10.1371/journal.pone.0216617

**Published:** 2019-05-13

**Authors:** Yinjunjie Zhang, Zhicheng Xu, Marco A. Palma

**Affiliations:** 1 Crawford School of Public Policy, Australian National University, Canberra, ACT 2601, Australia; 2 School of Economics, and the Center of Yellow River Civilization and Sustainable Development, Henan University, Kaifeng, Henan Province 475001, People’s Republic of China; 3 Department of Agricultural Economics, Texas A&M University, 2124 TAMU, College Station, TX 77843-2124, United States of America; Universitat Jaume I, SPAIN

## Abstract

The rapid development and diffusion of new technologies such as automation and artificial intelligence makes life more convenient. At the same time, people may develop overdependence on technology to simplify everyday tasks or to reduce the level of effort required to accomplish them. We conduct a two-phase real-effort laboratory experiment to assess how external assistance affects subsequent revealed preferences for the convenience of a lower level of effort *versus* monetary rewards requiring greater effort. The results suggest that men treated with external help in the first phase tend to choose more difficult options with potentially higher monetary rewards. In contrast, after being treated with external help, women exhibit a stronger propensity to utilize the convenience of an easier task and are less likely to choose a more difficult option that carries higher potential earnings.

## Introduction

Artificial intelligence and smartphones integrate multiple features to facilitate everyday life to the point where many people are becoming addicted to their use [1]. Today, remembering a telephone number or using a map to navigate to our destination are skills that are becoming obsolete. Middle school students develop dependency for information technology and the Internet to do their homework [2]. External help is not limited to technology. Helicopter parents provide excessive help to their children, who might consequently develop a dependency on their parents for doing almost everything. Nevertheless, there is also evidence that external help may have positive outcomes. For example, contrary to popular belief, a meta-analysis shows that the use of calculators improves mathematical operational and problem solving skills [3]. This paper aims to understand how the increasing reliance on external help may impact society.

Although economists have been interested in studying human behavior related to “help”, the focus has been on people’s willingness to offer help (e.g., altruistic behavior, charitable giving [4, 5, 6, 7, 8]). However, whether and how external assistance affects preferences and subsequent decision-making of the help recipients still remains an open question. The immediate benefits of receiving help are straightforward, but there may also be unintended consequences on subsequent behavior and performance. Motivated by the potential externalities of receiving assistance on the help recipients, we conduct a two-phase laboratory experiment to investigate how external assistance to a real-effort task in the first phase affects individual preferences for trading-off effort *versus* monetary rewards in a subsequent task. The potential effects of receiving help may impact future behavior and performance in two opposite ways. Individuals may use external help to boost their confidence and motivation to complete a task independently and even pursue more difficult tasks in the future. Meanwhile, it is also possible that the convenience from a lower level of effort —of receiving help— may erode human capital and crowd out intrinsic work ethic. Namely, people may develop a stronger dependency on the external help, reducing their willingness to learn new skills and take on more difficult challenges. Determining the outcomes of external help is important for evaluating the welfare effects of business management strategies and policy interventions designed to provide people with external assistance.

We pay particular attention to potential gender differences in reaction to external help. Gender composition is unbalanced in many fields, ranging from industry and politics to academia. There is ample evidence in the economics literature of significant gender differences in risk attitudes and competition. Relevant to our study, a large body of literature originating in psychology, documents substantial gender differences in the consequences of receiving help (see the next section for a comprehensive discussion). If men and women also experience differential impacts from external assistance, we believe it is critical to understand whether these asymmetric effects increase or reduce the prevalent gender gap.

Our laboratory experiment consists of two stages. Participants were randomly assigned to the treatment or control group. Subjects in the control group performed a paid real-effort task without any assistance, while subjects in the treatment group performed the same task with external assistance, receiving hints for the right answers that simplified the task significantly. The second stage introduced a different real-effort task. In order to elicit the subjective relative evaluation of monetary rewards against the convenience of external assistance (i.e., less effort), before the second task began, subjects were allowed to choose a payment schedule and effort level through a multiple price list (MPL) [9, 10]. The MPL offered subjects an array of ordered scenarios (in rows) that differed in potential earnings and the amount of external help. For each row, subjects had to choose between option A, with 16 questions (accordingly lower potential earnings) and option B, with 24 questions (higher potential earnings). External help was again provided as hints that simplified the task. The number of hints in option A decreased for each row of the MPL (from 16 to 0), while option B had a fixed number of hints (8). Consequently, the row in which a subject switched from option A to B provides a measure of his/her preference for the trade-off between the convenience of using external help and the extra effort required to obtain higher earnings (see the Appendix for a more detailed illustration).

***The first hypothesis is that being treated with external assistance in the first stage can influence an individual’s trade-off between monetary rewards and their dependence on external assistance by working on a task exerting less effort***. Further, our experimental design tests whether the treatment effects drive subjects to develop a behavioral dependency on its convenience or boost their confidence and motivation, leading them to perform the real-effort task with less external assistance in the subsequent task. Based on previous findings in the psychology literature related to reactions to help, we expect significant heterogonous responses across genders. ***The second hypothesis hence predicts that receiving external assistance has different treatment effects for female and male participants***.

The results show that, after being treated with help, men tend to overestimate their cognitive capability and underestimate the effort required to perform the real-effort tasks. Although there are no differences in performance by gender, men are overconfident and less likely to use external help. Women, on the other hand, exhibit a stronger propensity to utilize the convenience and choose a less challenging task in the second stage. We further explore the underlying mechanism of how cognitive bias affects individuals’ behavior by looking at differences in the switching patterns of the treatment and control groups conditional on the performance level.

## Related literature

Using external help as the treatment links our study to an abundant literature in psychology examining “reactions to help.” A review of literature helps to understand the roots of our findings. Fisher et.al. [11] argue that the effects of help are mixed, inducing either self-threatening or self-supportive experiences for the help recipients. On one hand, receiving help may hurt self-esteem by implied inferiority, inadequacy, and dependency. On the other hand, help can also be perceived as positive and supportive, often resulting in material gains [12].

Reactions to help differ by gender. The “threat to self-esteem” model suggests that when men receive help from a person with similar experience, it lowers their self-confidence. However, help can also provide stronger self-confidence if the giver has more experience [13, 14, 15]. Receiving help does not seem to harm the self-esteem and performance of women [16, 17]. Women are more inclined to admit that they need assistance and appreciate the help, while men experience more self-doubt. In our experiment, we find that after being treated with help, men have a stronger propensity to demonstrate their confidence by choosing a more challenging option, while women tend to develop greater dependency on the convenience of lower effort. It is noteworthy that in the above-mentioned research in psychology, the experimental design deliberately leads subjects to believe that their performance is a reflection of their intellectual abilities. In most cases researchers also lead participants to believe that they performed significantly worse than their peers. In order to avoid contamination from potential self-doubt and negative feelings, we did not provide subjects negative or positive feedback about their performance until the end of the experiment.

There is a small but growing literature in experimental economics that discusses gender differences in responding to external advice. For instance, Brandts et al. [18] point out that external advice from an experienced person has different impacts on men and women’s work efficiency and competition entry in a real-effort task. They mainly focus on the impacts of external advice on the decision to enter a tournament, while our experiment examines the extent to which external assistance can affect confidence and effort in a subsequent task. Heikensten and Isaksson [19] examine how the gender of the advisors influences individuals’ advice-seeking decision and whether this impact is heterogeneous across genders. While they focus on the gender of influencers, our design concentrates on the influencees’ willingness to receive subsequent help after a training session and the potential gender differences from them. Notably, a major difference between previous studies and ours is that the external help in our experiment is provided by a *computer* rather than another person. Hence, the results of our experiment are more suitable for understanding the effects of non-human help.

Our experiment also mirrors a large literature on gender differences in risk preferences and competition [20, 21, 22, 23, 24, 25]. A notable finding in this literature is that men and women have remarkable differences in their propensity to engage in competitive behaviors. To be specific, women shy away from competition, while men are more competitive, even in tasks in which they are not more capable than women [26, 27, 28]. In field experiments of intellectual [29] and physical competition [30], men show greater effort and better performance in a competitive environment, while women’s performance remains unchanged regardless of the environment’s competitive level.

More closely related to the findings in our study, previous research suggests that men seem to gain self-esteem by demonstrating that they are better than others [31, 32, 33]. In contrast, Günther et.al. [34] find that women avoid competing with men, even in areas where women wrongly believe they have lower performance. Niederle and Vesterlund [26] show that about one third of the gender gap in tournament entry can be explained by gender differences in confidence. Therefore, our experiment is complimentary to studies on gender differences related to over-confidence and self-esteem. Note that these stylized findings may not be entirely driven by innate gender-specific characteristics. Women’s under-performance in competitive environments also depends on the task [27, 34, 35, 36], the gender composition of the competing group [29, 30, 37], stereotype and information conditions [38], and cultural and social norms (e.g., patriarchal society vs. matrilineal society) [39]. Overconfidence may be useful to explain our experimental result showing that after receiving help, men have greater willingness to take the challenge of a more difficult task.

Our experiment differs from previous literature in that most past studies have compared gender differences in competition with other people. In contrast, our study focuses on gender differences in the reaction to external help arising mainly from technology dependence. Although our results provide useful insights into the impact of technology dependence on daily life and labor market participation, the latter seems to be receiving increasing attention from economists [40, 41].

## Experimental design and method

**Fig 1:**
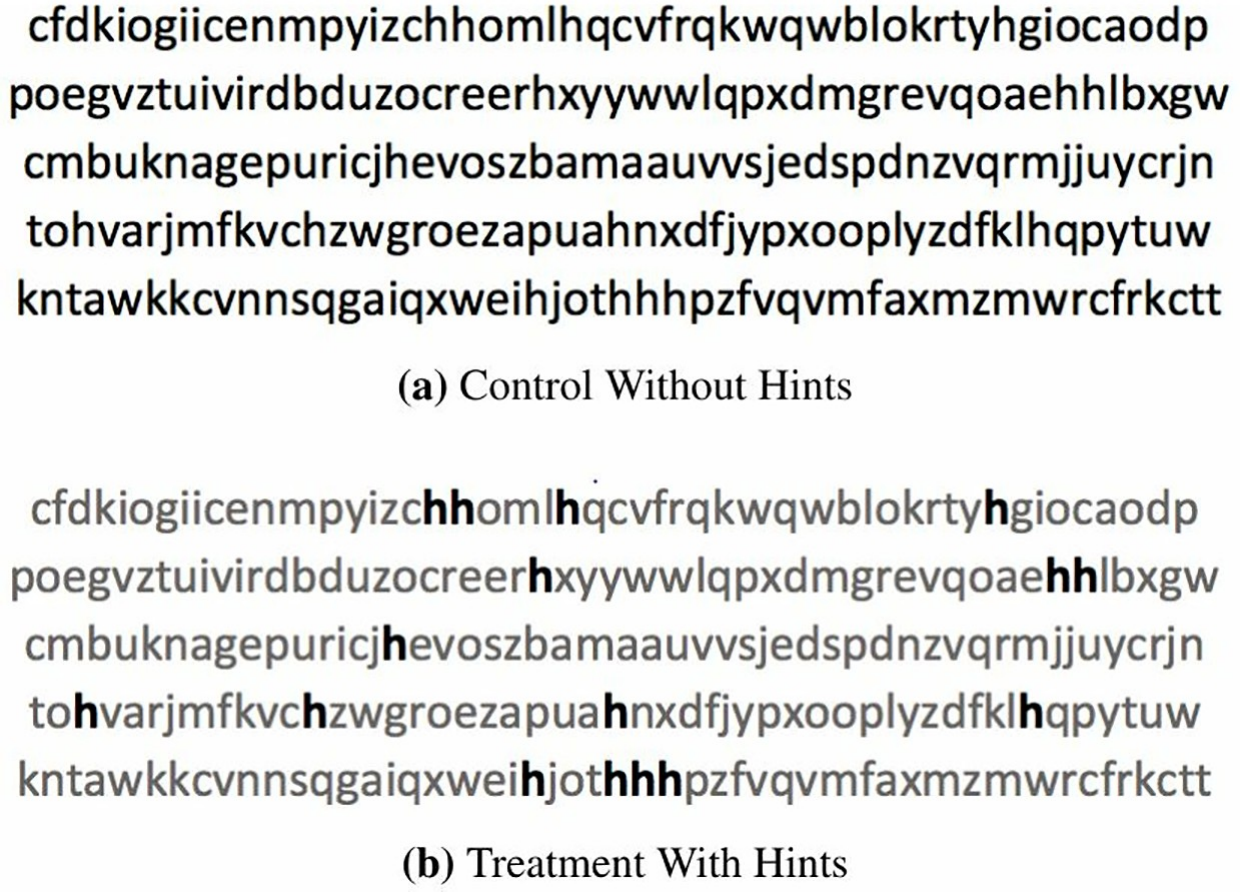
An example of the real-effort task in the first stage.

The experiment has two stages. The first stage is a real-effort task consisting of ten questions. Participants were asked to count the number of times a predetermined letter appeared in a text with five lines of random letter combinations. Each participant had an equal chance of being randomly assigned to the *treatment* group, where they would work with the hints, or to the *control* group, where they would work without hints. In the treatment, participants were provided with external help in the form of hints which made all irrelevant letters less salient—although they were still present—to simplify the counting task (see Fig 1).

Before proceeding to the ten questions, all subjects viewed the same sample of two questions, one with hints and another one without hints, and then a lottery immediately followed to decide whether the subject was assigned into the treatment or control group. A timer displayed in the right corner of the screen counted the time used for each question which gave participants a sense of effort level to complete the task.

Over the course of the first-stage task, subjects were not allowed to proceed to the next question until they provided the correct answer. Participants were allowed multiple attempts to enter the right answer to each question, and they had to correctly answer all ten questions to complete this stage. As such, all participants earned $10 at the end of the first stage, in which we avoid the potential income effects. We hypothesize that the hint treatment in this stage would influence an individual’s preference of trading off payment for receiving external help in the subsequent stage.

**Fig 2:**
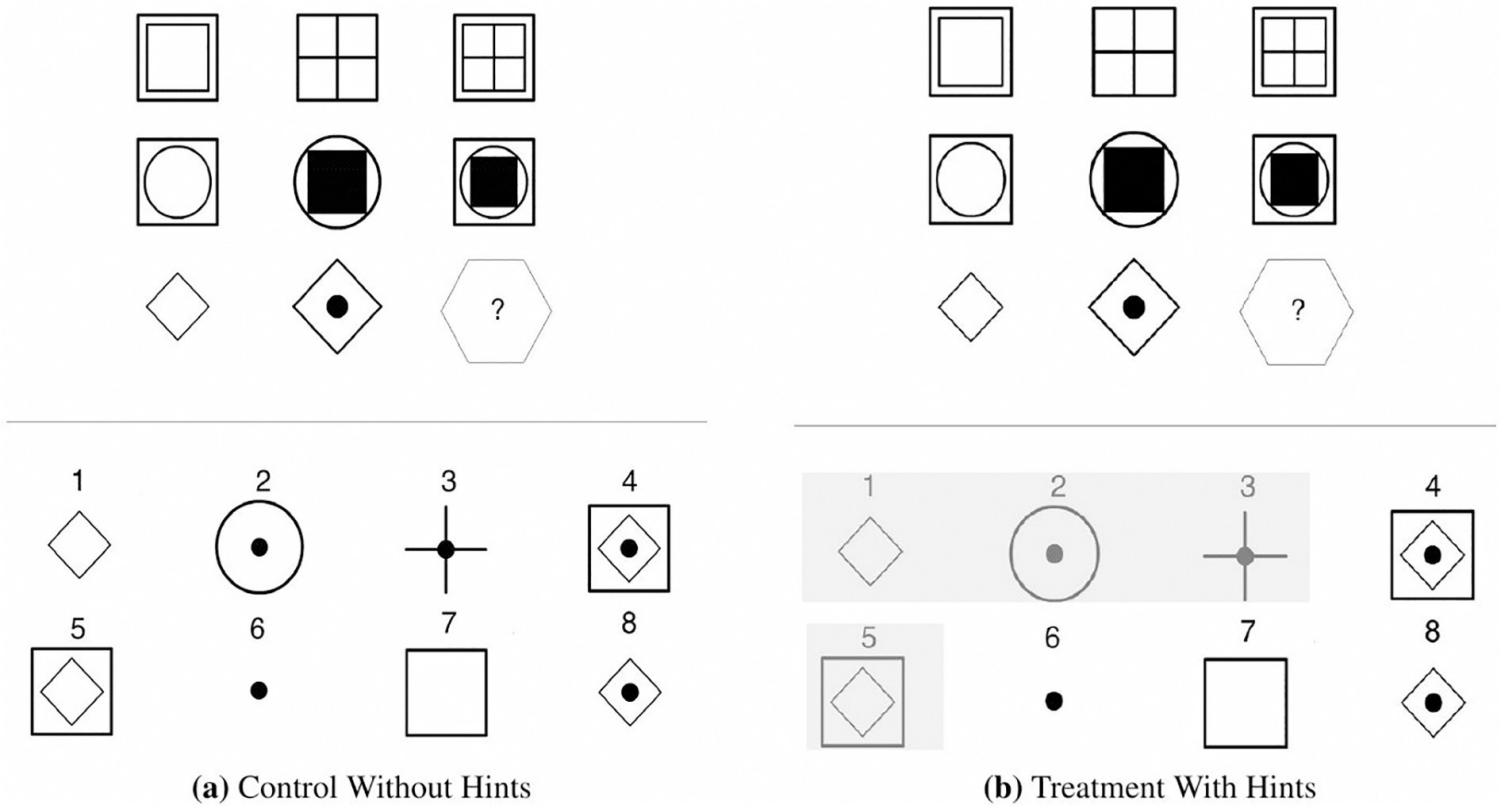
An example question from the task of Raven’s Test.

In the second stage, each subject was randomly assigned to either another real-effort task or a Raven’s test. Again, participants were provided with sample questions before performing the task. The real-effort questions in this stage were very similar to those in the previous one, with the only difference that the second stage used numbers instead of letters. An example of a Raven’s test question is shown in Fig 2. Analogous to the real-effort case, the hints suppressed some irrelevant answers helping individuals to reduce the answer pool.

The goal of using two types of task in this stage was to detect whether behavioral patterns induced in the first stage would be significantly adjusted due to the similarity of the tasks between the two stages. Mann-Whitney *U* tests of the key indicators show no significant differences between the two types of tasks.

**Fig 3:**
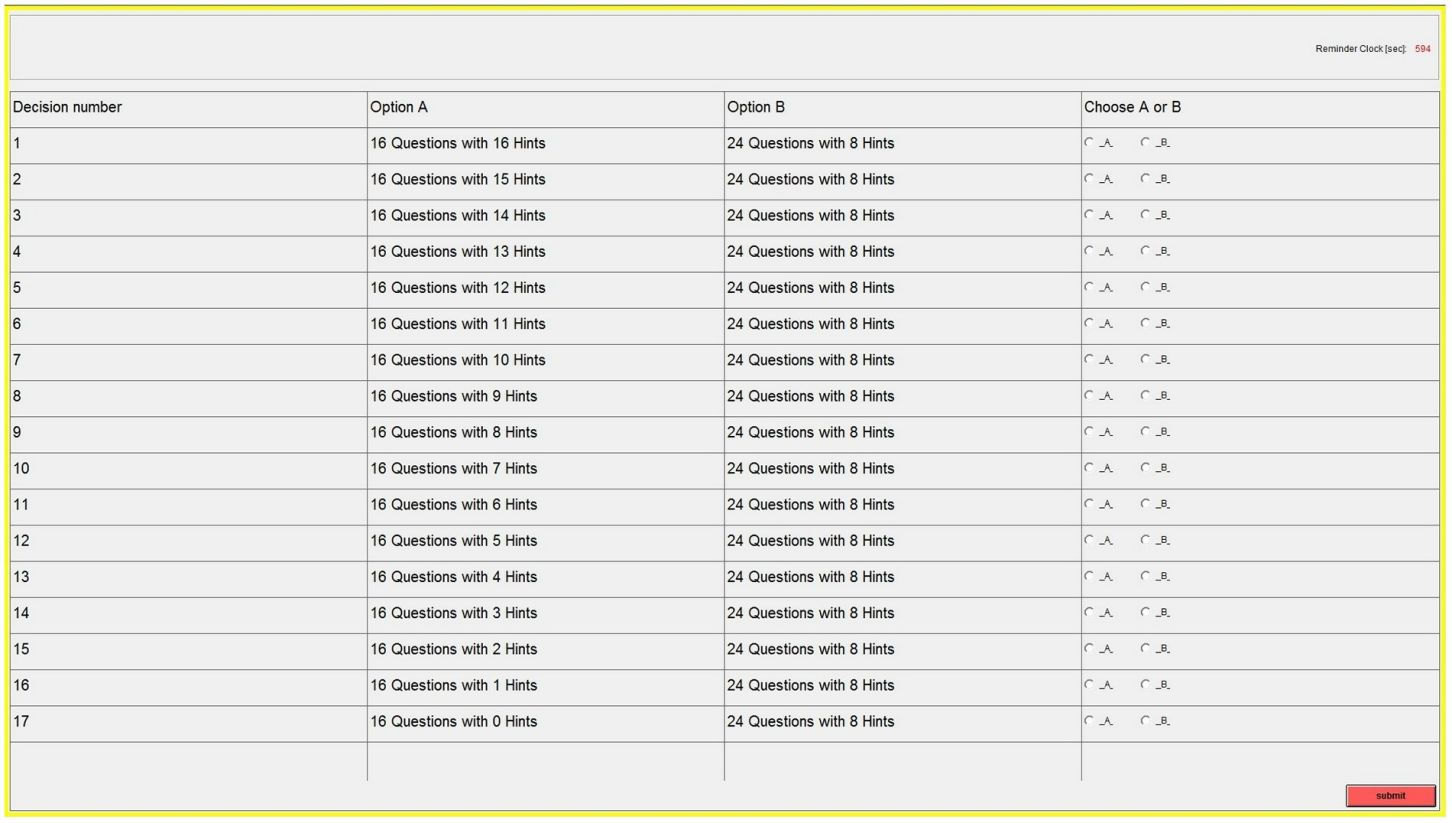
Binary choices in the multiple price list.

Our elicitation of preferences for external help and monetary rewards is through a multiple price list (MPL) mechanism. This is shown in Fig 3. Before participants starting the second stage task, they were invited to select option A or B in each of the 17 choice sets.

Option A had 16 questions and option B had 24 questions; hence option B always has higher potential earnings. The number of hints in option A is descending in the list from 16 in the first row to 0 in the last row, while the number of hints in option B is fixed at 8. Note that the attractiveness of option A decreases by each decision row. This can be easily illustrated by comparing the first two rows in Fig 3. In the first row, subjects face a trade-off between option A, which would pay $16 with a very high probability (16 questions and 16 hints), while option B has higher possible payoff because of more questions (24) but also requires greater effort because of fewer hints (8) under the same time limit. In the second row, option A becomes less attractive compared to the first row as with the same number of questions (16) there are fewer hints (15). Accordingly, the subjects’ willingness to select option A diminishes as the row number increases. The row number in which a subject switches from option A to B provides a measure of individual preferences for monetary rewards over external help. We argue that preferences are influenced by the hint treatment introduced in the first stage. The earlier a subject switches from option A to B, the more evident that the subject is willing to forgo external help and choose a more difficult task with higher potential earnings. (See the Appendix section for a more sophisticated analysis).

Subjects were informed that a lottery at the second stage would randomly determine the choice pair to be realized, and the realized choice set will apply to all participants in the session. Each individual therefore will work on the option chosen in the realized one choice pair. Throughout the ten sessions, in total about 38% of participants end up working on option A and 62% on option B.

All participants had 20 minutes to complete the second stage task, and each correct answer was worth $1. In order to collect earnings, subjects cannot make errors in more than 25% of the questions. This payment criterion was to discourage subjects from always choosing option B based on strategic thinking. Since option B always has a higher potential payoff than option A, participants would have a higher chance of earning more money by choosing option B if there is no restriction on the accuracy rate. This rule also increased the salience of external assistance by raising the difficulty level of option B.

We collect a set of variables on individual characteristics in the end of the experiment. These include gender, race, religion and ideology. In particular, we elicit individuals’ subjective evaluation on performance by inviting them to guess their ability rank compared to the peers in the second stage. This was done before notifying them about their actual earnings. The selection is formatted using a scale from zero to ten, where ten represents better than all others, and zero is no better than any other. As the second stage task is administered to complete in 20 minutes, this belief elicitation on performance is mainly based on subjects’ inference about their accuracy rate in the second stage task.

The experiment was computerized using the software ‘z-Tree’ [42] and conducted at Texas A&M University. This study was approved by the Internal Review Board from Texas A&M University—Office of Human Research Protections (OHRP), Human Subjects Protection Program. The approval identification number is IRB2016-0313D. All participants in the study had provided written, informed consent before the study began, and the data analysis was conducted anonymously.

We used a between-subject design and each subject participated in only one session with the duration about 60 minutes, including sign-up, consent, decision making, and payment. Before entering the laboratory, participants were informed of receiving a $5 show-up fee upon completing the tasks and having the opportunity to earn extra payoffs based on their decisions and performance. However, they were not provided with any details about the experiment. A figure explaining the sequence of the experiment is attached in the Appendix (see S1 Fig).

## Results

We assume that rational agents have only one switching point from option A to option B during the MPL stage. The uniqueness of the switching point is proven by the theoretical framework provided in Appendix A. After excluding 17 subjects who made multiple switches, a total of 160 subjects remain in the sample.

Our research question is to examine whether the treatment of external assistance affects individuals’ subsequent behavior through the induced cognitive bias, and whether the prediction of heterogeneous responses across genders found in the psychology literature also persists in our setting with incentivized economic decisions.

To answer that we first need to exclude the possibility of sample selection bias. A balance check of the sample is presented in Table 1. The *t*-tests report that there are no significant differences between the treatment and control groups over a set of demographic covariates.

**Table 1:** Balance check.

Variables	Control	Treatment	*p*-value
Age	22.72(.46)	23.31(.41)	0.342
Male	0.47(.06)	0.45(.05)	0.784
Undergraduate	2.66(.05)	2.54(.06)	0.123
Ideology to right(1-5)	2.91(.14)	3.08(.13)	0.367
Belong to a religion org.	0.32(.05)	0.34(.05)	0.866
Family income			
<$15,000	0.12(.04)	0.12(.04)	0.944
($15,000, $35,000)	0.30(.05)	0.30(.05)	0.973
($35,000, $60,000)	0.14(.04)	0.20(.04)	0.306
($60,000, $100,000)	0.14(.04)	0.11(.03)	0.514
> $100,000	0.30(.05)	0.27(.05)	0.639
Obs.	77	83	

Notes: Standard errors are reported in parentheses. *p*-values are reported for two-side *t*-tests. Mann-Whitney U tests report similar results.

We then test if the hint treatment in the first stage serves the purpose of reducing the required effort to complete the task. Table 2 compares the average time spent per question in the treatment with that in control groups during the first stage. As seen, introducing hints substantially improved the performance of both genders in the treatment group, spending significantly less time per question. The spent time difference in treatment and control groups presents an objective measurement of the convenience provided by the external help. The first stage training also gives participants a reference point to make their switching choices in the MPL stage.

**Table 2:** Time spent per question in the first stage.

	Control	Treatment	*p*-value
All subjects	63.85(2.16)	19.61(1.04)	0.000
	N = 77	N = 83	
Male	62.47(3.22)	19.95(1.73)	0.000
	N = 36	N = 37	
Female	65.07(2.94)	19.35(1.27)	0.000
	N = 41	N = 46	
*p*-value	0.552	0.775	

Notes: Time spent is measured in seconds. Standard errors are reported in parentheses. *p*-values are reported for two-side *t*-tests. Mann-Whitney U tests report similar results.

We introduce two types of tasks in the second stage: another real-effort task similar to the first stage and a Raven’s test. The purpose of this is to detect whether behavioral patterns induced in the first stage, if any, will be significantly adjusted based on the similarity of the tasks over the two stages. Mann-Whitney *U* tests (Table 3) of the key indicators—including switching patterns, subjective beliefs on ability, accuracy rate per question, and time spent per question—show no significant differences between the tasks. This indicates the induced treatment effect on switching patterns is not related to differences in the type of tasks.

**Table 3:** Task type combination comparison.

	Effort + Effort	Effort + Raven	*p*-value
Switch point	8.78(4.95)	8.24(5.20)	0.733
Self-evaluation in the 2^*nd*^ stage	6.06(1.94)	6.11(1.68)	0.945
Time spending per question in the 1^*st*^ stage	39.15(26.74)	42.05(26.67)	0.330
Time spending per question in the 2^*nd*^ stage	36.06(9.93)	36.45(11.10)	0.789
Percent of correct answers in the 2^*nd*^ stage	0.74(.18)	0.72(.15)	0.325
Obs.	63	97	

Notes: Standard deviations are reported in parentheses. *p*-values are reported for two-side Mann-Whitney U test. *t*-tests report similar results. Self-evaluation measures the self-reported evaluation of individual second stage performance compared to the rest of the participants in the session. 10 = better than 100% of others, 0 = no better than any others. Time is measured in seconds.

In the following section, we further evaluate the effect of second stage task type using the fixed effect model under the difference-in-differences (DD) framework. It shows that our results are robust and not affected by the task types in the second stage. Next, we pool the data of the two types of tasks in the analysis.

### Heterogeneous treatment effects on revealed preference

We show the overall comparison of the switching point over the two experimental groups in the first row of Table 4. Subjects who received external help in the first stage do not show significantly different switching patterns compared to the control group. On average, both groups switched from option A to B between the 8th and 9th decision row. Further, it shows that the first-stage treatment affected the two genders’ switching patterns in opposite ways, offsetting the overall effect.

**Table 4:** Switching point comparison.

	Control	Treatment	*p*-value
All subjects	8.68(.60)	8.24(.55)	0.592
	N = 77	N = 83	
Male	9.50(.77)	6.81(.85)	0.022
	N = 36	N = 37	
Female	7.95(.89)	9.39(.67)	0.194
	N = 41	N = 46	
*p*-value	0.198	0.018	

Notes: Standard errors are reported in parentheses. *p*-values are reported for two-side *t*-tests. Mann-Whitney U tests report similar results.

Heterogeneous responses over genders in reaction to help have been documented in psychology literature, although the conclusion was unsettled. Our experiment provides insights into this question under the economic setting. Therefore, it is worth to briefly summarize this heterogeneity before we formally test it in an econometric model.

**Fig 4:**
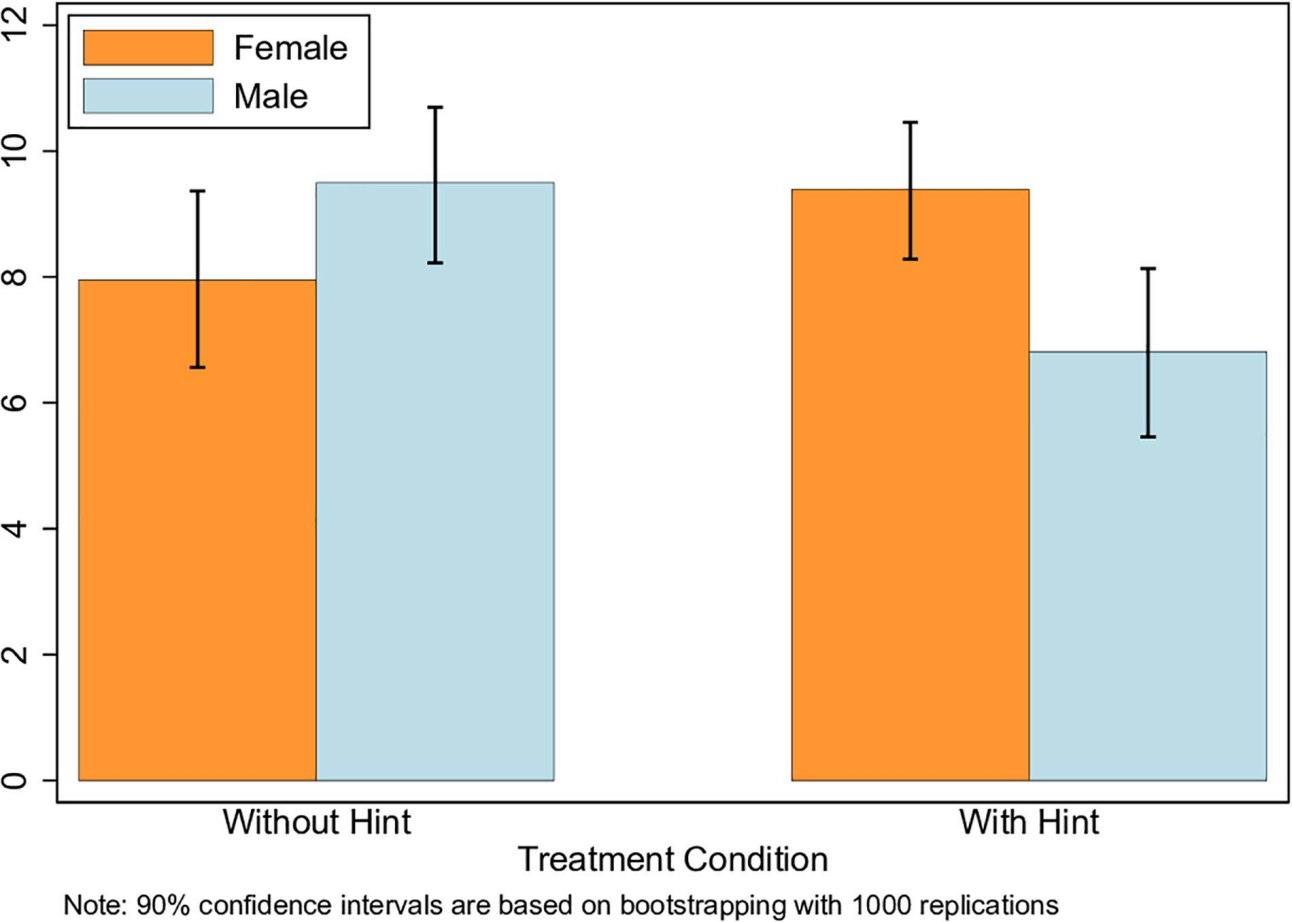
Switching point gender comparison by bootstrap method.

As seen in Table 4, treated men switched earlier than men in the control group. Controlled male subjects, on average, made the switching decision between the 9th and 10th question, while treated male subjects switched around the 6th and 7th question. This difference is significant at the 5% level. In contrast, women tend to be reluctant to switch too early if they received hints in the first stage, although the difference is subject to large variation. As the switching points present with multiple peaks and skewed distributions, we check the robustness of our findings by building confidence intervals using the bootstrapping method (Fig 4).

**Fig 5:**
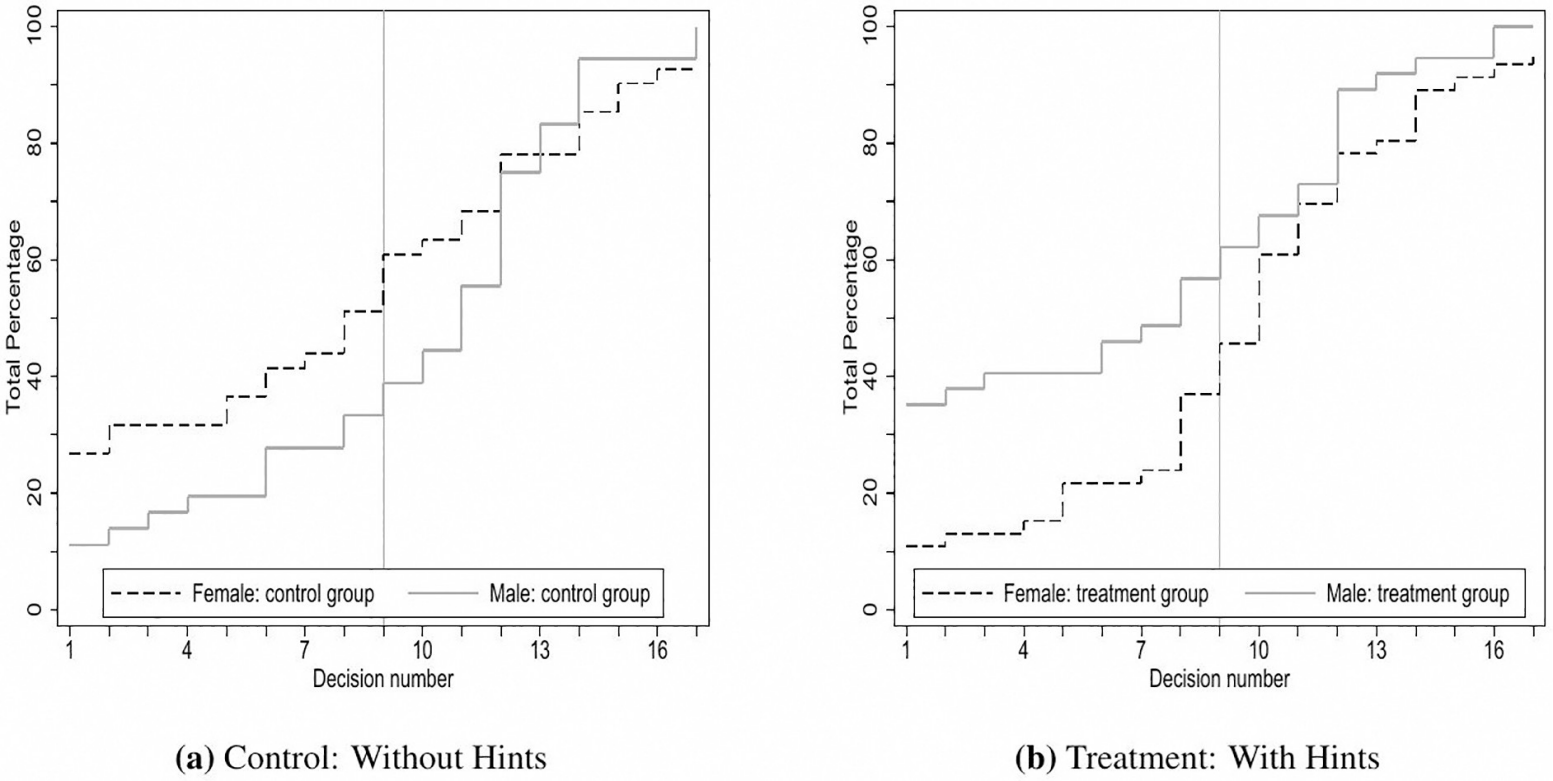
Cumulative switching point from option A to B.

Fig 5 displays the cumulative percentage of switching points at each decision row in the control and treatment groups. In both graphs, gender differences are more pronounced at the beginning, but gradually disappear at the end of the MPL.

For the control group, up until the 12th decision row, women’s cumulative percentage of switching points is always higher than men’s (panel a of Fig 5). Half of the females assigned into the control group in the first stage switched before the 8th decision row, while this ratio for males is less than 35%. Men closed the gap with women by the 13th decision row, where nearly 80% of both genders have switched. For the rest of the decisions (14th–17th), the cumulative percentage of switching points for men is slightly higher than women. This suggests that, without the external help treatment, women seem to place a higher value in the potential monetary payout compared to males by making the trade-off in favor of option B (with carries a higher possible payment) in the initial part of the MPL. Meanwhile men tend to avoid a higher level of effort by switching later than women.

Interestingly, this pattern is reversed with the external help treatment (panel b in Fig 5). Compared to the control group (without hints), women’s switching points were significantly delayed, while men switched much earlier. (In our theoretical model, this indicates that after being treated with hints, *α* decreases for women but it increases for men, see the Theory section in the appendix.) Over 35% of male participants receiving hints in the first stage chose to switch to option B at the first row of the MPL, compared to only 11% of females. More than 60% of male participants switched by the 9th decision row, compared to only 45% of females. Women closed the gap with men at around the 11th decision row. The distinctive change in the switching pattern by gender again indicates that men are more likely to switch later in the control, but they are more likely to switch earlier in the treatment. Meanwhile, a significant number of treated females converged to switching between the 8th and 12th decision row.

We formally test this relationship in the following difference-in-differences framework:

$$\text{Switching}\;{\text{point}}_{ist}=\omega Female_{i}+\theta Hint_{t}+\delta \left(Hint_{t}\cdot Female_{i}\right)+\gamma_{s}+X_{ist}\beta +\epsilon_{ist},$$ (1)

where *γ*_*s*_ is session fixed effects, *Female*_*i*_ is gender indicator for subject *i*, and *X*_*ist*_ captures individual characteristics (see Table 1). *Hint*_*t*_ ⋅ *Female*_*i*_ is the interaction of external help treatment and the gender indicator, which is equal to 1 for female participants assigned to the treatment group and 0 otherwise. The parameter ***δ*** is our key difference-in-difference estimator.

The point estimates are reported in Table 5. We implement the estimations by gradually adding controls for fixed effects of session or task type, and individual characteristics such as background covariates, self-evaluations and ability. As per our design, we proxy individual ability using the performance in the second stage (proportion of correctly answered questions). We argue that individual ability is unlikely to be affected from the first stage to the second stage of the experiment, particularly since both tasks are very similar. Recall that the MPL lottery did not evenly allocate participants over the two options of realized choice pair, in the most complete specification we also control for option assignment.

**Table 5:** Difference-in-difference estimates of gender gaps in switching points.

	(1)	(2)	(3)	(4)	(5)
	Switching Point	Switching Point	Switching Point	Switching Point	Switching Point
Hint Treatment	-2.689**	-2.735**	-3.952***	-4.036***	-2.996***
	(1.146)	(1.155)	(1.345)	(1.308)	(1.084)
Female	-1.549	-1.953*	-2.490*	-1.920	-1.086
	(1.175)	(1.147)	(1.270)	(1.394)	(1.113)
Hint Treatment*Female	4.129**	4.272***	5.804***	5.357***	4.369***
	(1.599)	(1.620)	(1.692)	(1.739)	(1.402)
Constant	9.500***	9.702***	6.110	7.442	10.711*
	(0.767)	(0.791)	(7.167)	(7.301)	(5.642)
Session Fixed Effects	No	Yes	Yes	No	No
Task Fixed Effects	No	No	No	Yes	Yes
Demographic Variables	No	No	Yes	Yes	Yes
Observations	160	160	160	160	160

Notes:

* *p* < 0.1,

** *p* < 0.05,

*** *p* < 0.01 Robust standard errors are reported in the parentheses.

In the third and fourth column, we further control the self-reported evaluation and ability (proxied by the second stage performance) to exclude any confounders from individual confidence. In the last column, we additionally include into the control variables the option distribution of the realized decision in MPL. Tobit estimation (not reported here) censoring at the switching point between 0 and 17 yields similar point estimates across the three specifications.

As seen, in response to the treatment of external help, female participants on average switched 4–5 decision rows later than male counterparts. The point estimates are statistically significant at the 5% level in column (1) and at the 1% level as we controlling for session fixed effects in columns (2) and (3), task type fixed effects in column (4), and option distribution in the last column. In particular, point estimates through specification (3) and (4) suggest using two different types of tasks (Real-effort task and Raven’s test) in the second stage does not affect the robustness of result, which is in line with the previous analysis presented in Table 3. Column (5) indicates the realized distribution of the shares of participants working on different choice options do not affect the estimate either.

### Exploring the potential causes for the gender gap in reaction to external assistance

Thus far, our results have shown that the first stage hint treatment drives men and women to differ substantially in their switching patterns in the subsequent MPL stage. While male participants appear to place a higher value in the monetary payout, female participants seem to value more the convenience of external help. Next, we attempt to explore the possible mechanisms through which the hint treatment causes these divergent effects.

#### Performance in the second stage

We use two ways to compare the objective performance over the two genders in the second stage, i.e., performance in aggregate level and by question. As shown in Table 6, panel a reports the proportion of correct answers in the second stage overall and by gender. Although male subjects are willing to take a more difficult option, their performance is not different from that of females. This indifference holds even when the sample is divided by treatment assignment or by the number of questions selected in the second stage. In panel b, we compare the time spent per question (in seconds). Again, there are no significant differences by gender.

**Table 6:** Gender differences in the second stage performance.

	Female	Male	*p*-value
*Panel a: share of correctly answered questions*			
Pooled	0.74(.02)	0.72(.02)	0.521
	N = 87	N = 73	
Control	0.75(.03)	0.73(.03)	0.589
	N = 41	N = 36	
Treatment	0.72(.03)	0.71(.02)	0.693
	N = 46	N = 37	
16 Questions	0.81(.03)	0.74(.04)	0.140
	N = 33	N = 27	
24 Questions	0.70(.02)	0.71(.02)	0.604
	N = 54	N = 46	
*Panel b: time spending per question*			
Pooled	35.24(1.16)	37.57(1.21)	0.167
Treatment	34.15(1.66)	38.14(1.38)	0.077
Control	36.45(1.60)	36.99(2.02)	0.835
16 Questions	33.07(2.40)	38.14(2.45)	0.148
24 Questions	36.56(1.14)	37.24(1.29)	0.695

Notes: Standard errors are reported in parentheses. *p*-values are reported for two-side *t*-tests.

Mann-Whitney U tests report similar results. Time is measured in seconds.

In appendix S2 and S3 Figs, we further present the cumulative distribution of the proportion of correct answers for the overall sample and by treatment conditions. At each performance level, it depicts the share of individuals who solve at most that proportion of correct answers in the second stage. Across all the graphs, the distributions closely track each other. It is unlikely that gender differences in ability drive the heterogeneous treatment effects.

#### Cognitive bias

**Fig 6:**
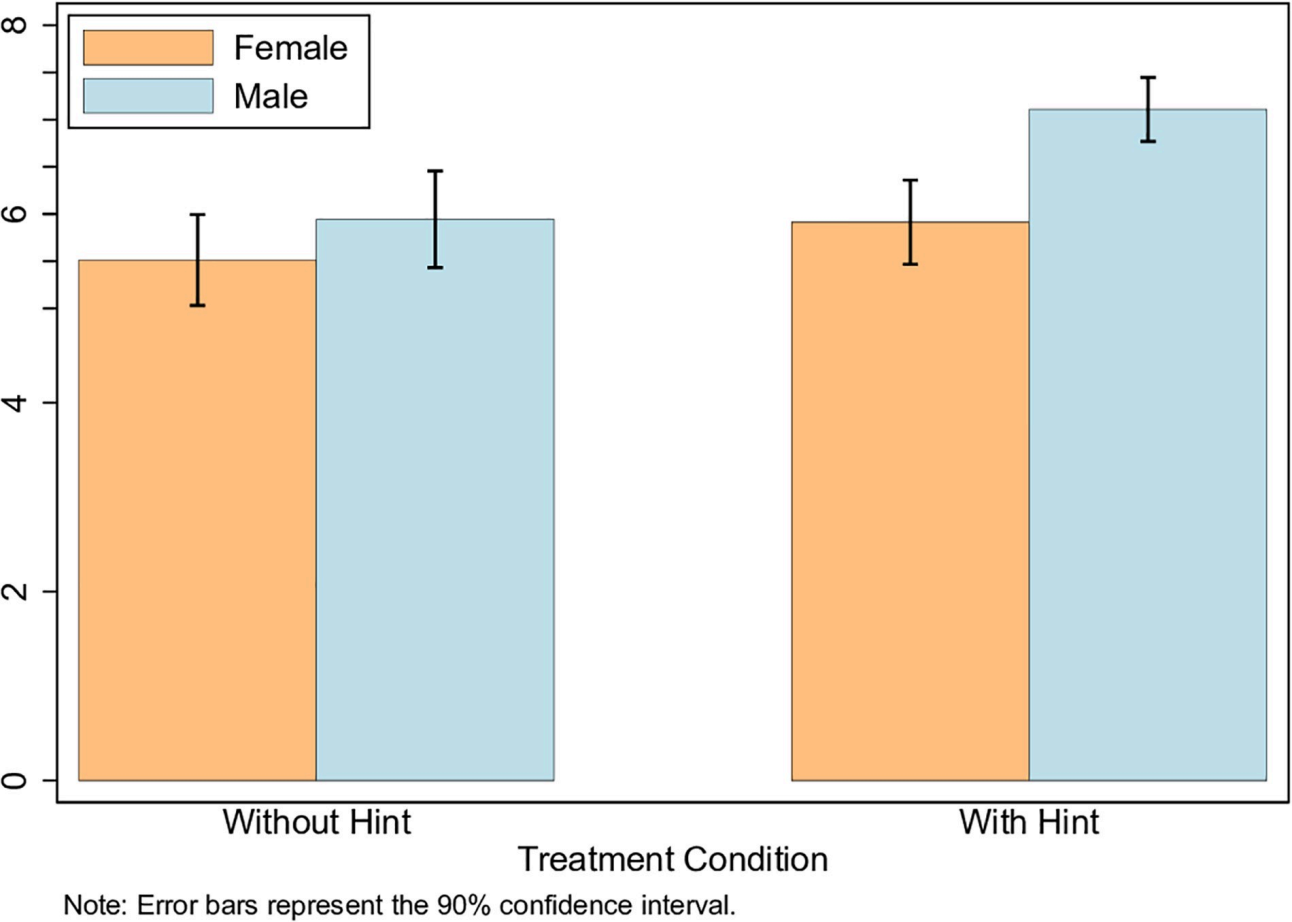
Self-evaluation of second stage performance by gender.

We then test whether differences in individuals’ cognitive biases about their ability drive the results. At the end of the second stage and before being notified about their earnings, participants were asked to report their beliefs regarding their performance relative to others. Fig 6 shows the mean gender comparison of self-evaluated performance. (Subjects are asked to evaluate their performance relative to the rest of the participants in the same session. 10 = better than 100% of others, 0 = no better than any others.) The two-sided *t*-test suggests that there are no significant gender differences in the control group (*p* = *0.303*). However, there is a significant difference in the treatment group (*p* = *0.001*). A difference-in-difference estimation showed similar results (Table 7). Clearly, the treatment significantly boosted the confidence and subjective beliefs of men, despite no significant differences in the actual performance between genders (Table 6).

**Table 7:** Difference-in-difference estimates of gender gaps in self-reported performance.

	(1)	(2)	(3)
	Self-evaluation	Self-evaluation	Self-evaluation
Hint Treatment	0.214	0.267	0.269
	(0.372)	(0.381)	(0.384)
Male	0.415	0.509	0.498
	(0.413)	(0.462)	(0.464)
Hint Treatment*Male	1.144**	1.159**	1.181**
	(0.522)	(0.538)	(0.540)
Constant	5.529***	4.210**	4.206**
	(0.281)	(1.625)	(1.622)
Session Fixed Effects	Yes	Yes	Yes
Demographic Variables	No	Yes	Yes
Obs.	160	160	160

Notes:

* *p* < 0.1,

** *p* < 0.05,

*** *p* < 0.01.

Notes: Robust standard errors are reported in parentheses. Self-evaluation measures the self-reported evaluation of individual second stage performance compared to the rest of the participants in the session. 10 = better than 100% of others, 0 = no better than any others. In the third column, we additionally include into the control variables the option distribution of the realized decision in MPL.

The greater confidence shown by men provides suggestive evidence that cognitive bias could be driving the earlier switching patterns exhibited by men. In contrast, women’s self-evaluation on performance did not significantly change by the treatment. Women’s late switching is not driven by changes in their beliefs about own ability. While receiving the treatment induces men to become overconfident, regardless of their true ability, they significantly underestimate the required effort to complete the task.

One alternative explanation on the potential channel of behavioral change could be a differential feeling of perceived luck between the two experimental groups. Obviously, individuals assigned to the control group might feel less lucky than those in the treatment group. Men and women may have different feelings of luck resulting from hints. This may be a channel that interprets the gender differences in treatment effect, although it is not a confounding factor against our hypothesis on the gender-specific treatment effect. We admit that it is worth exploring how the perception of luck interact with men’s overconfidence after receiving the external help, we have to leave this question open for future work. Other possible mechanisms behind the results are examined in appendix.

## Conclusion and discussion

Everyday, people appear to rely more on external help from new technologies. In this laboratory experiment, we focused on the effects of external help on the trade-off between higher potential monetary rewards requiring greater effort and the convenience of lower effort from external assistance.

In particular, we find that after receiving help men tend to overestimate their cognitive ability and underestimate the necessary effort to perform a real-effort task. Consequently, men are more likely to choose a more difficult task with higher potential earnings after being treated with external help, but they do not perform better than women.

Women, on the contrary, tend to adjust their beliefs and decisions based on external supporting information in the opposite way. We argue that although external help may induce weak-performing women to utilize the convenience of external assistance, the possibility of strong-performing women to also develop a dependency cannot be ruled out (see the Appendix B for an elaborate analysis).

To some extent, the observed treatment effect differences by gender may be useful to explain why women are more risk averse and avoid competition, while men actively engage in competitive behavior. According to our results, external assistance makes women more likely to depend on it. This, in turn, might drive women to behave more conservatively. The behavioral bias exhibited by men indicates a refusal to external help, which ultimately becomes financially costly by reducing their earnings (see the Appendix B).

## Supporting information

S1 Fig Flow chart of the experimental sequence. (TIFF)

S2 Fig CDF of the proportion of correctly solved problems in the second stage: Pooled sample. (TIFF)

S3 Fig CDF of the proportion of correctly solved problems in the second stage: Treatment vs. control group. (TIFF)

S4 Fig Switching point conditional on the performance in the second stage: Treatment vs. control group. (TIFF)

S5 Fig Share of positive profits in the second stage conditional on performance: Treatment vs. control group. (TIFF)

S6 Fig Share of positive profits in the second stage by treatment. (TIFF)
