# Supplementary figures and images for: Conveniently dependent or naively overconfident? An experimental study on the reaction to external help
Source: PLoS One. 2019 May 13;14(5):e0216617. doi: 10.1371/journal.pone.0216617 (PMC6513094; doi:10.1371/journal.pone.0216617)

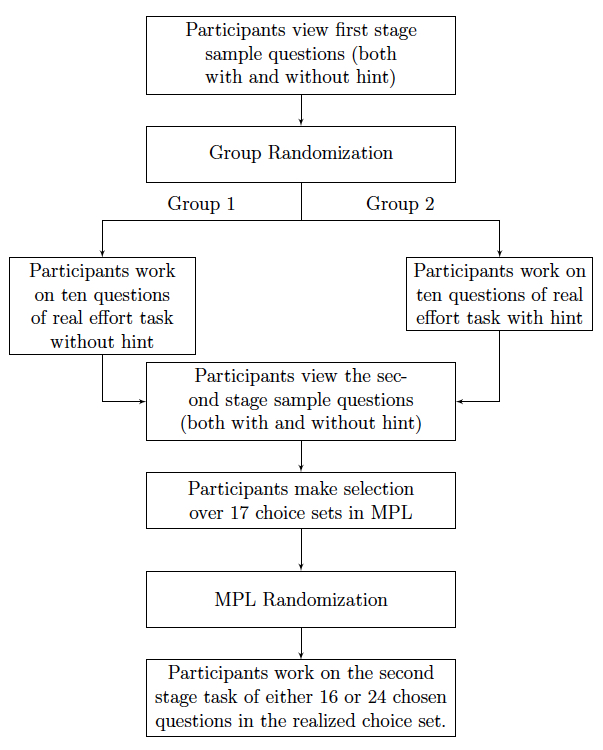

Supplement: S1 Fig — (TIFF) [file pone.0216617.s001.tiff]

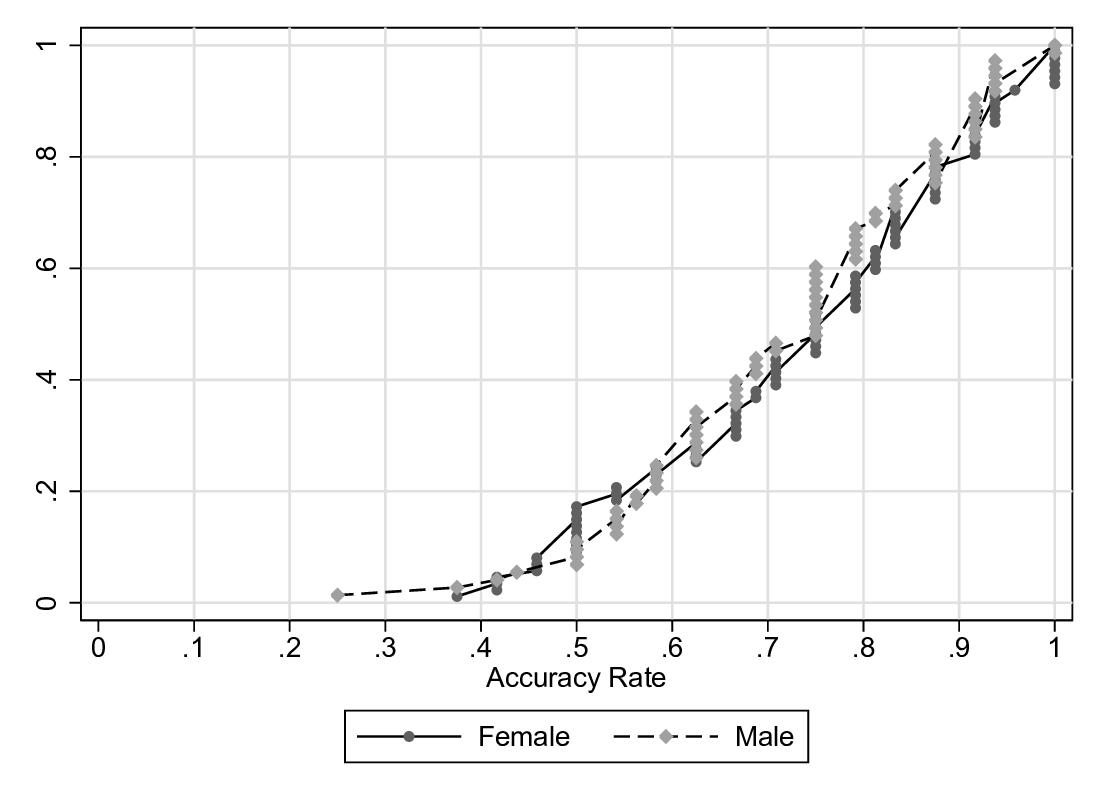

Supplement: S2 Fig — (TIFF) [file pone.0216617.s002.tiff]

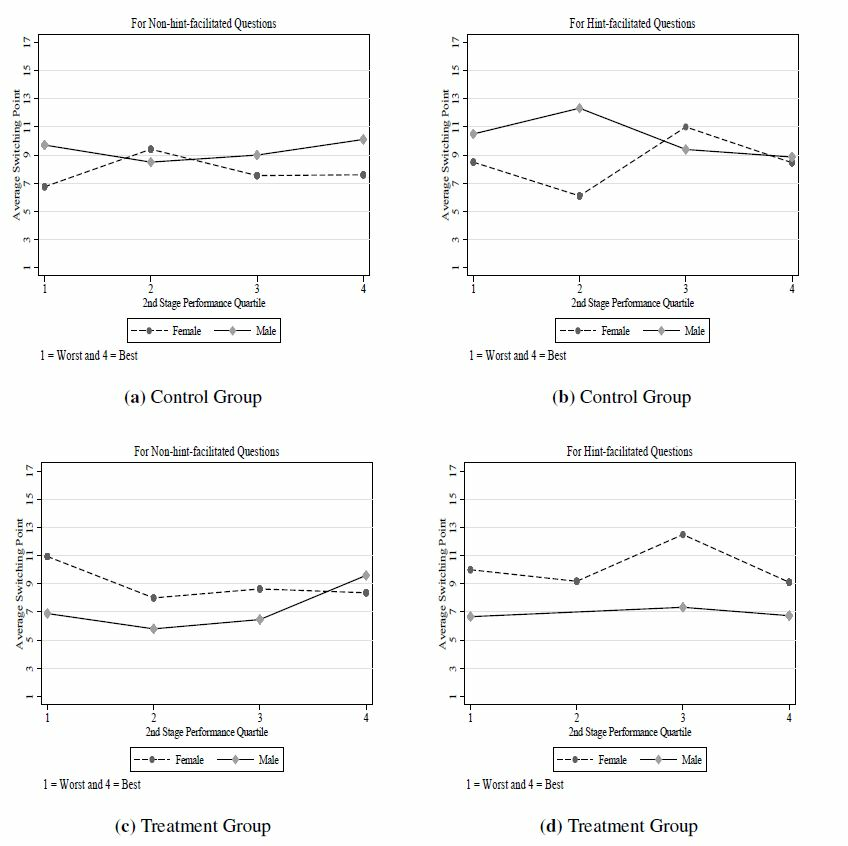

Supplement: S4 Fig — (TIFF) [file pone.0216617.s004.tiff]

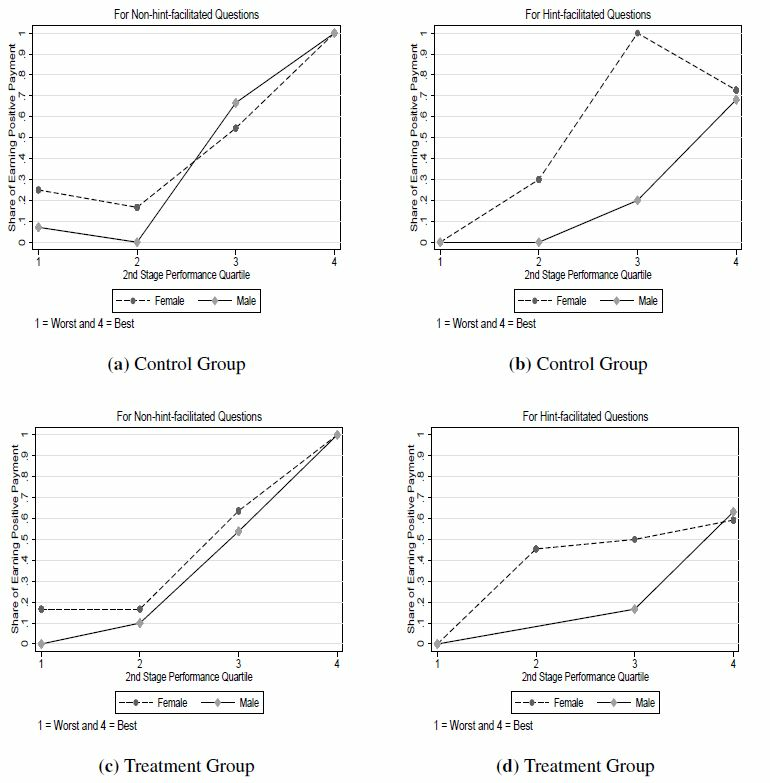

Supplement: S5 Fig — (TIFF) [file pone.0216617.s005.tiff]

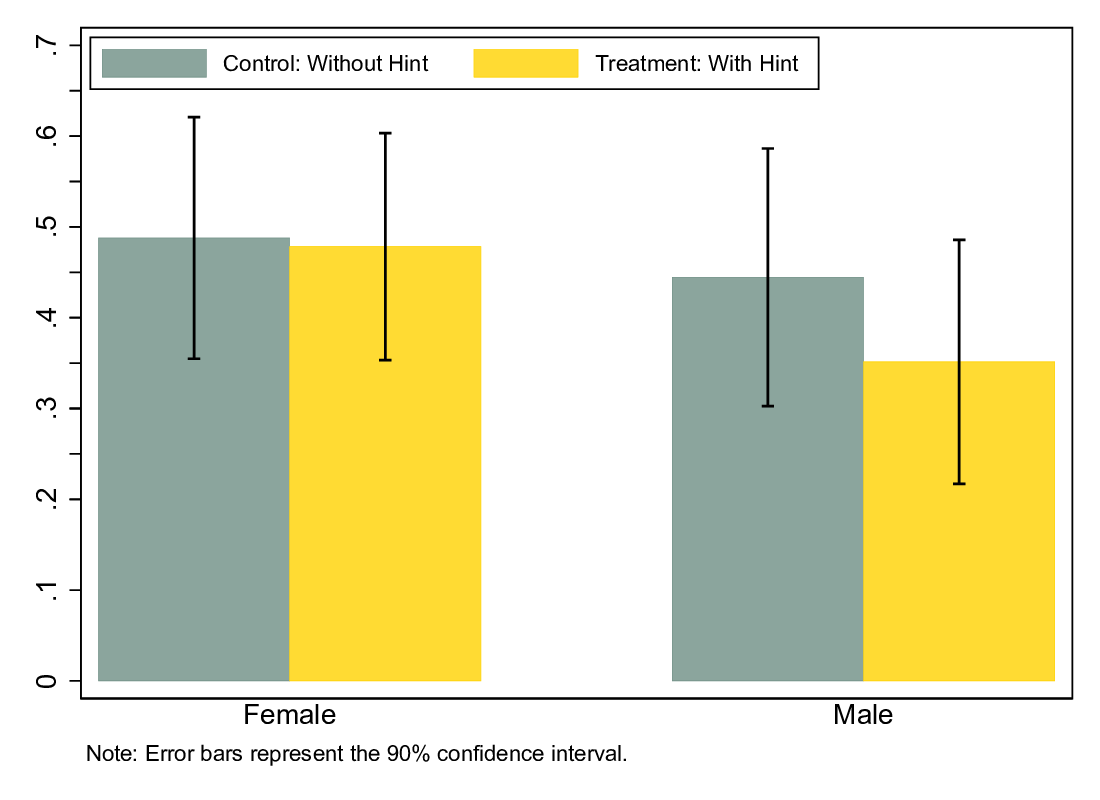

Supplement: S6 Fig — (TIFF) [file pone.0216617.s006.tiff]
